# Loss of IP_3_ receptor function in neuropeptide secreting neurons leads to obesity in adult *Drosophila*

**DOI:** 10.1186/1471-2202-14-157

**Published:** 2013-12-18

**Authors:** Manivannan Subramanian, Siddharth Jayakumar, Shlesha Richhariya, Gaiti Hasan

**Affiliations:** 1 National Centre for Biological Sciences, Tata Institute of Fundamental Research, Bangalore 560065, India; 2 Mysore University, Mysore 570006, India; 3 Manipal University, Manipal 576104, India

**Keywords:** Calcium, Lipid homeostasis, Hyperphagia, *Magro*

## Abstract

**Background:**

Intracellular calcium signaling regulates a variety of cellular and physiological processes. The inositol 1,4,5 trisphosphate receptor (IP_3_R) is a ligand gated calcium channel present on the membranes of endoplasmic reticular stores. In previous work we have shown that *Drosophila* mutants for the IP_3_R (*itpr*^*ku*^) become unnaturally obese as adults with excessive storage of lipids on a normal diet. While the phenotype manifests in cells of the fat body, genetic studies suggest dysregulation of a neurohormonal axis.

**Results:**

We show that knockdown of the IP_3_R, either in all neurons or in peptidergic neurons alone, mimics known *itpr* mutant phenotypes. The peptidergic neuron domain includes, but is not restricted to, the medial neurosecretory cells as well as the stomatogastric nervous system. Conversely, expression of an *itpr*^*+*^ cDNA in the same set of peptidergic neurons rescues metabolic defects of *itpr*^*ku*^ mutants. Transcript levels of a gene encoding a gastric lipase *CG5932 (magro),* which is known to regulate triacylglyceride storage, can be regulated by *itpr* knockdown and over-expression in peptidergic neurons. Thus, the focus of observed *itpr* mutant phenotypes of starvation resistance, increased body weight, elevated lipid storage and hyperphagia derive primarily from peptidergic neurons.

**Conclusions:**

The present study shows that *itpr* function in peptidergic neurons is not only necessary but also sufficient for maintaining normal lipid metabolism in *Drosophila*. Our results suggest that intracellular calcium signaling in peptidergic neurons affects lipid metabolism by both cell autonomous and non-autonomous mechanisms.

## Background

Calcium is a key signaling molecule in multi-cellular organisms that regulates a variety of cellular processes [1,2]. The IP_3_R (Inositol 1,4,5 trisphosphate Receptor) is a ligand gated calcium channel present on the membranes of endoplasmic reticular (ER) stores. It mediates the release of ER calcium upon binding of its cognate ligand IP_3_. In *Drosophila* there is a single gene, *itpr,* for the IP_3_R which is 60% homologous to mammalian IP_3_R1 [3]. Previous studies have shown that expression of the *Drosophila* IP_3_R is widespread in all tissues and cell types examined [4,5]. However, depending on their allelic strength, *itpr* mutants exhibit relatively specific metabolic and neuronal phenotypes. Hetero-allelic combinations of strong *itpr* mutants exhibit metabolic defects, altered feeding and transcriptional changes in metabolic gene pathways during larval stages [6,7]. *itpr*^*ka1091*^ and *itpr*^*ug3*^ are point mutations in the modulatory domain (Gly 1891 Ser), and in the ligand binding (Ser 224 Phe) domain respectively of the IP_3_R. These mutants are lethal as homozygotes, while their hetero-allelic combination (*itpr*^*ka1091/ug3*^ or *itpr*^*ku*^) is adult viable [8]. Recently, we demonstrated the presence of metabolic changes in *itpr*^*ku*^ adult animals leading to starvation resistance, increased body weight, elevated TAGs and hyperphagia [9].

In mammals, disorders like type 2 diabetes, coronary heart disease, respiratory complications and osteoarthritis are a result of altered fat metabolism [10]. The complexity of these diseases arises in part from regulation of fat metabolism through the interaction of signaling pathways involving multiple tissues and organs. Genetic studies in model organisms help understand aspects of this complexity. In *Drosophila*, fat metabolism is essential for maintaining energy homeostasis. Nutrient fat in the form of Triacylglycerides (TAGs) is broken down to fatty acids in the mid-gut, absorbed and re-synthesized as TAGs in the fat bodies [11]. Perturbations in fat metabolism can lead to changes in TAG levels and consequent obesity [11,12]. Lipids stored in fat body cells are utilized under stress conditions and the storage and mobilization of lipids to target tissues is tightly regulated, based on energy requirements. This requires communication between the gut, fat body cells and oenocytes, the cells analogous to the mammalian liver in *Drosophila*[13]. Furthermore, signals from the brain coordinate feeding behavior as well as the utilization of stored TAGs, finally affecting the body weight of an organism [14-16]. Based on the obese and hyperphagic phenotypes of *itpr*^*ku*^ it appears that calcium release by the IP_3_R helps maintain this axis of lipid metabolism and feeding in *Drosophila*. Here we show that peptidergic neurons are an important focus of IP_3_R function in the context of metabolic control.

## Results

### The IP_3_R affects *Drosophila* metabolism through its function in peptidergic neurons

**Figure 1:**
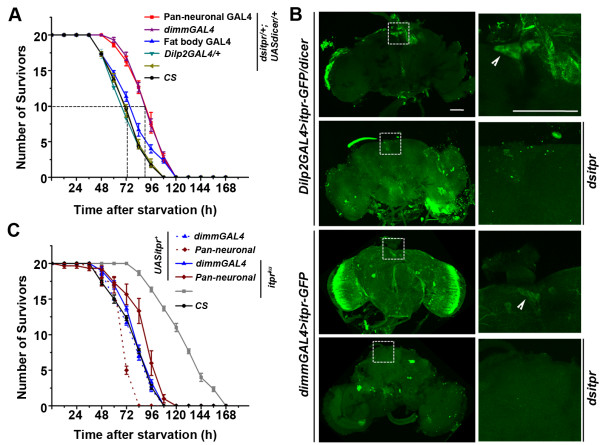
***itpr *levels in peptidergic neurons are critical for the starvation resistance phenotype. (A)** Flies with knock down of *itpr* using pan-neuronal and *dimm* drivers show starvation resistance as compared to the wild type whereas *itpr* knock down in either Dilp2 neurons or fat bodies do not show any starvation resistance. **(B)** Flies with knock down of *itpr* in Dilp2 neurons and *dimm* neurons using *UASitpr-GFP* showed decrease in GFP levels when compared to their controls without knockdown, cell bodies shown by arrow heads. Scale bar is 50 μm. **(C)** Over-expression of *itpr*^*+*^ in both pan-neuronal and peptidergic domains rescues starvation resistance observed in *itpr*^*ku*^.

To identify the tissue focus of *itpr* mutant phenotypes an *itpr* RNAi strain (*dsitpr*) was used to specifically knockdown the IP_3_R in all neurons and in the fat body. Pan-neuronal knockdown of the IP_3_R lead to a significant level of starvation resistance. In contrast, animals with knock down of the IP_3_R in the fat body exhibited the same extent of viability post starvation as control animals (Figure 1A). Obesity, starvation resistance and hyperphagia in *itpr*^*ka1091/ug3*^ mutants can be rescued by expression of an *itpr*^*+*^ cDNA in a subset of peptidergic neurons that secrete the insulin-like peptides (Dilps) amongst other neuropeptides [9,17,18]. These cells are marked by the *Dilp2GAL4* strain. To test for necessity of the IP_3_R in Dilp neurons, *dsitpr* was driven by *Dilp2GAL4*. Surprisingly, these animals did not exhibit any starvation resistance (Figure 1A). Consequently, we tested animals with knockdown of *itpr* by the *dimm GAL4* that expresses in a larger subset of exclusively peptidergic neurons (including the Dilp neurons) [18]. This resulted in animals with a significant level of starvation resistance when compared with controls (Figure 1A).

In order to test if lack of starvation resistance with IP_3_R knockdown in Dilp2 neurons, arose due to insufficient knockdown, we expressed a GFP tagged IP_3_R transgene (*UAS-itprGFP*) in both Dilp2 neurons and peptidergic neurons marked by *dimmGAL4.* These strains were tested for the extent of GFP fluorescence in the presence and absence of IP_3_R knockdown by *dsitpr*. GFP expression was abrogated in the DILP neurons with both drivers indicating efficient knock-down (Figure 1B). Thus, IP_3_R knockdown is essential in multiple peptidergic neurons for generating starvation resistance. Concurrently, when an *itpr*^*+*^ cDNA was over-expressed in either all neurons or peptidergic neurons in the background of *itpr*^*ku*^, the rescued animals displayed a viability profile similar to that of controls (Figure 1C).

**Figure 2:**
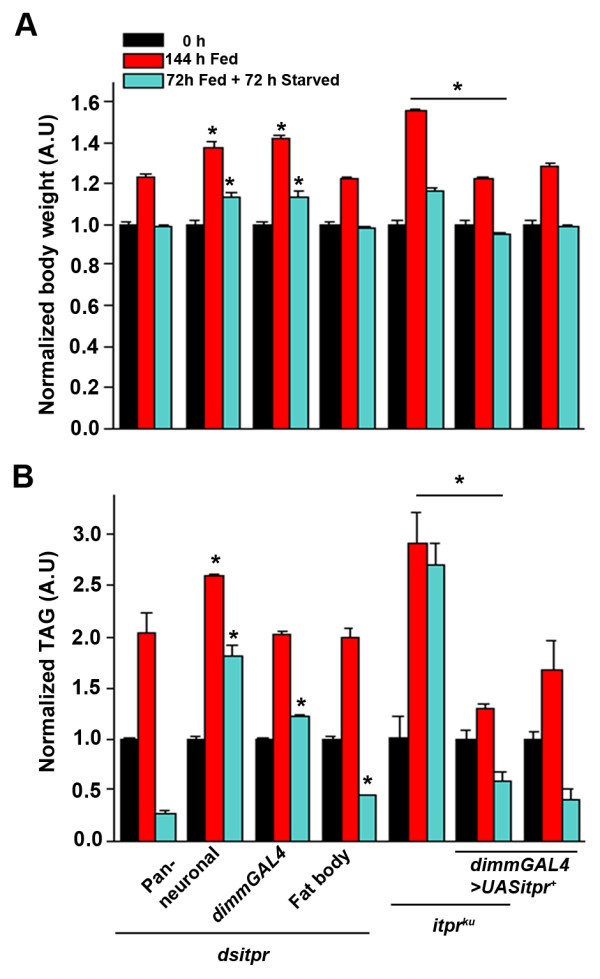
***itpr *function in neurons regulates body weight and fat storage under starvation. (A)** Flies with pan-neuronal and peptidergic knock down of *itpr* show a significant increase in their body weights post feeding for 144 hrs as well as post starvation for 72 hrs as compared to the RNAi heterozygote control. Over-expression of *itpr*^*+*^ in peptidergic neurons in the mutant background rescues body weight in both fed and starved conditions as compared to the mutant. The body weights are normalized to the respective 0 hrs time point for each genotype. **(B)** Quantification of TAGs from fly abdomens. Pan-neuronal knock down of *itpr* shows elevated levels of TAGs under fed and starved conditions when compared to the *dsitpr/+* control. Peptidergic knock down of *itpr* shows elevated TAGs post starvation as compared to the RNAi control. *itpr*^*+*^ over-expression using the *dimm* driver in the *itpr*^*ku*^ mutant background restores the TAG levels near wild type under both fed and starved conditions. The TAGs are first normalized to total protein and then to the respective 0 hrs time point for each genotype. *p < 0.05; Student’s *t* test; difference significant as compared to the corresponding time point of the RNAi control unless otherwise indicated.

Next we measured the body weights of animals with pan-neuronal and peptidergic knockdown of the IP_3_R. Similar to what has been observed for *itpr*^*ku*^ these knockdown animals showed significantly higher body weights after feeding for 144 hours. Body weights remained significantly elevated post-starvation for 72 hours. Knockdown of *itpr* exclusively in the fat body however, fails to elicit the above phenotype. Additionally, the elevated body weight of *itpr*^*ku*^ mutants (as first seen in [9]) was rescued by over-expression of *itpr*^*+*^ in peptidergic neurons (Figure 2A).

Pan-neuronal knock down of the IP_3_R also lead to elevated levels of TAGs in the abdomen at 144 hrs after feeding. Moreover, in flies with *itpr* knockdown using pan-neuronal and peptidergic GAL4s the TAG levels are high even after starvation for 72 hrs. The fat body knockdown of *itpr* animals also have slightly but significantly higher TAG levels post starvation (Figure 2B). This result agrees with the partial rescue of the elevated TAGs of *itpr*^*ku*^ by over expression of *itpr*^*+*^ exclusively in the fat body observed previously [9]. On the other hand, over expression of *itpr*^*+*^ in the peptidergic neurons alone completely rescues the elevated TAGs post feeding and starvation (Figure 2B). These data suggest that *itpr* function in peptidergic neurons is both necessary and sufficient for maintaining the level of stored TAGs in fat body cells of adult *Drosophila*.

### IP_3_R function in peptidergic neurons regulates food intake

**Figure 3:**
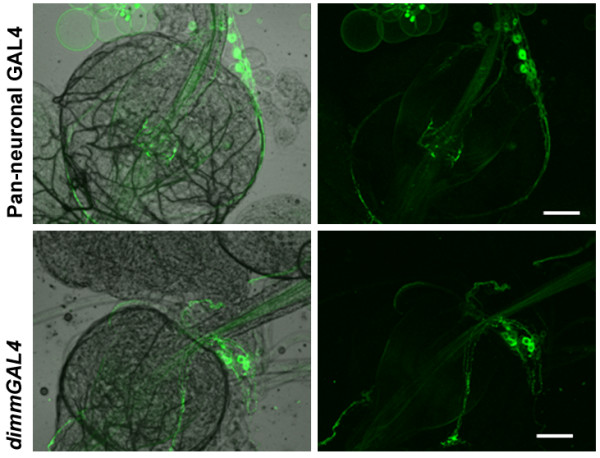
**Peptidergic neurons express in the proventriculus.** Neuronal cell bodies were observed on the larval proventriculus when mCD8-GFP was driven with both, the pan-neuronal (top) and peptidergic (bottom) drivers. Scale bar is 50 μm.

**Figure 4:**
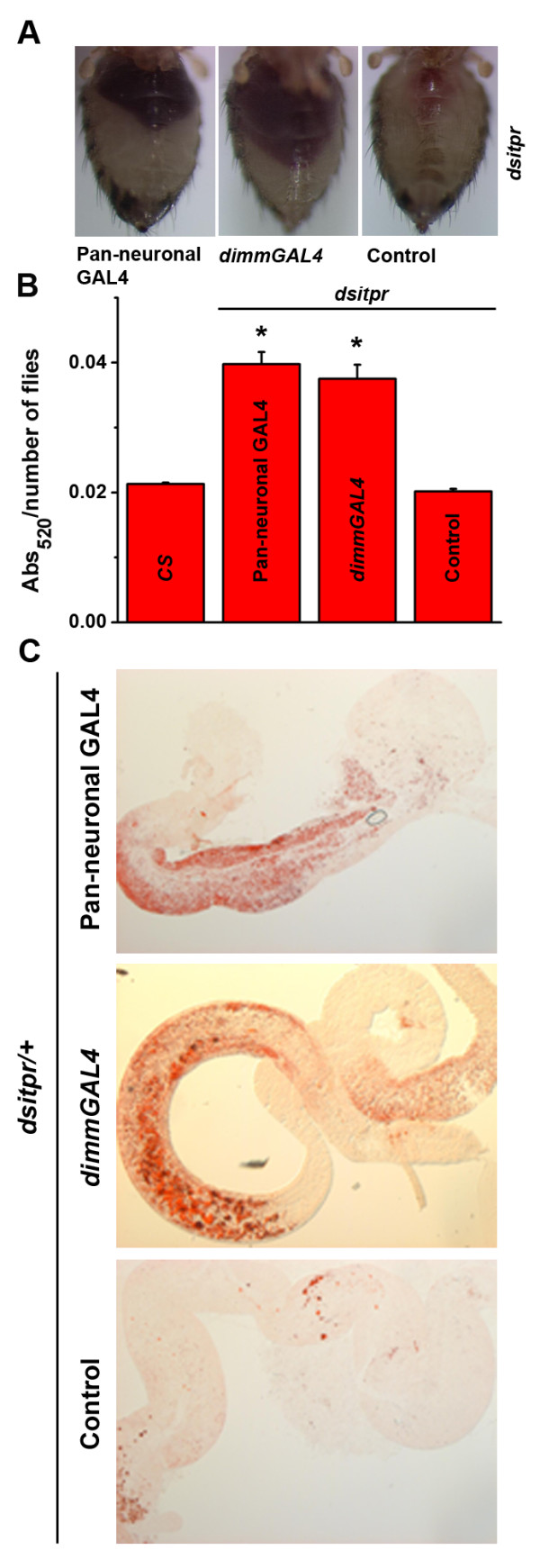
**Knockdown of *itpr *in neurons shows increased feeding. (A)** Flies with pan-neuronal and peptidergic *itpr* knockdown show increased feeding as compared to the RNAi control as observed in their abdomen when fed colored food. **(B)** Spectrophotometric quantification of the red dye in fly abdomen (*p < 0.05; Student’s *t* test; difference significant as compared to the RNAi control). **(C)** Oil Red O staining for TAGs in the gut also shows increased accumulation of TAGs in the guts of flies with pan-neuronal and peptidergic knock down of *itpr* as compared to the RNAi control.

**Figure 5:**
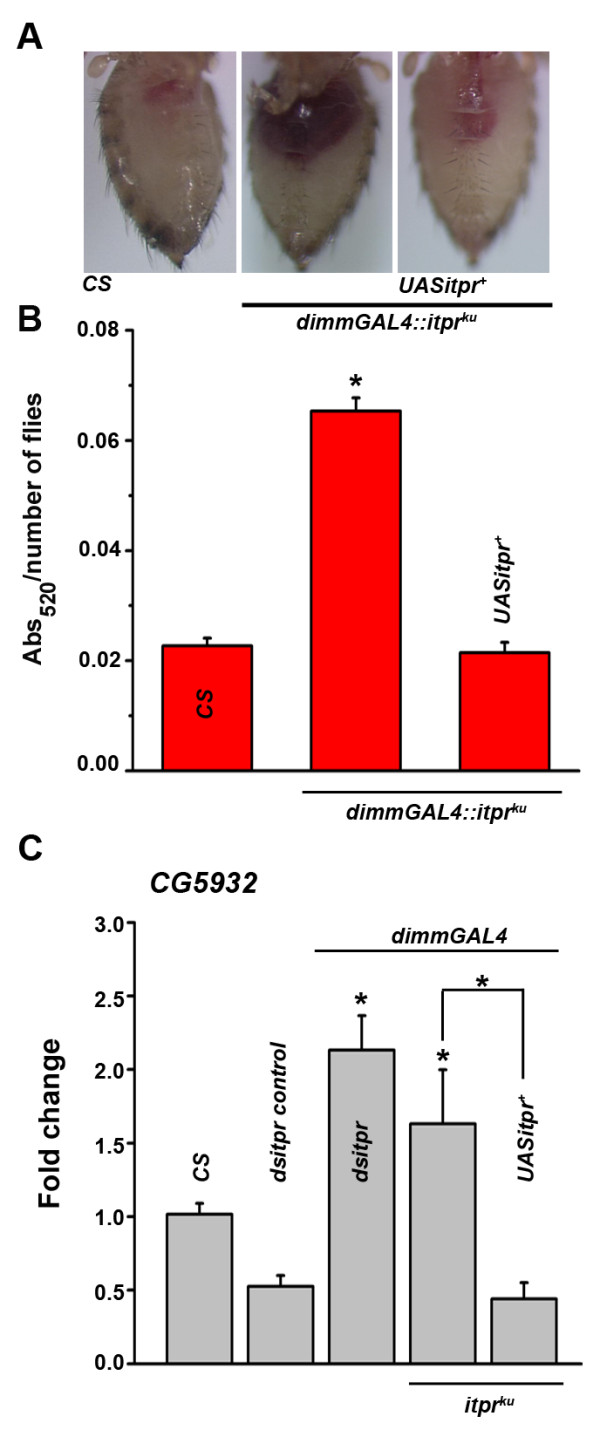
**Over-expression of *itpr***^***+***^**in peptidergic neurons rescues feeding defect of *itpr***^***ku***^**mutant. (A)** Over-expression of *itpr*^*+*^ in peptidergic neurons rescues the feeding defects of *itpr*^*ku*^ mutants as seen by the amount of red dye in their abdomen. **(B)** Spectrophotometric quantification of the red dye in fly abdomen. **(C)** Quantitative real-time PCR analysis of the transcript levels of gastric lipase *CG5932 magro* from gut tissue of peptidergic knock down of *itpr* is similar to that observed in *itpr*^*ku*^. Over-expression of *itpr*^*+*^ in the peptidergic neurons in the mutant background restores its levels to near wild type. *p < 0.05; Student’s *t* test; difference significant as compared to the wild type unless otherwise indicated.

Obesity of *itpr*^*ku*^ derives in part from excess feeding or hyperphagia [9]. Feeding behavior in *Drosophila* is regulated by neurons which innervate and control satiety signals in the gut [19]. The presence of peptidergic neuronal cell bodies on the proventriculus, the insect stomach, has been reported previously [18,20]. Similarly, we observed that both pan-neuronal GAL4 and the peptidergic GAL4 strains (*dimmGAL4*) used in our studies express in neurons that innervate the gut, with cell bodies on the proventriculus (Figure 3). To test if excess feeding observed in *itpr*^*ku*^ flies has a neuronal focus, animals with either pan-neuronal knockdown of the IP_3_R or with knockdown in peptidergic domains were tested for the quantity of food ingested within a fixed time period by including an edible red dye in their food. A significantly higher quantity of red dye was observed in abdomens of animals with either pan-neuronal or peptidergic neuron knockdown of the IP_3_R (Figure 4A, B). Oil red O staining demonstrated elevated TAGs in the guts of flies with IP_3_R knockdown indicating greater ingestion and digestion of lipids (Figure 4C). Next we tested if expression of *itpr*^*+*^ in peptidergic neurons rescued the excess feeding observed in *itpr*^*ku*^[9]. *itpr*^+^ expression in peptidergic neurons of *itpr*^*ku*^ rescued hyperphagia as evident from the significantly reduced level of red dye in the abdomens of rescued animals compared with control animals (Figure 5A, B).

### IP_3_R function in peptidergic neurons affects transcription of a gut lipase

For obese and hyperphagic animals, excess storage of TAGs is likely to be accompanied by altered levels of lipid metabolizing enzymes in the gut. Magro, encoded by *CG5932,* is the *Drosophila* homolog of mammalian gastric lipase, LipA and is expressed in the anterior part of the gut and released into the intestine to digest TAGs. Levels of Magro have been shown to modulate TAG levels as knocking down Magro shows reduction in TAG levels [21]*. itpr*^*ku*^ mutants have elevated levels of TAGs as well as elevated levels of *magro*[9]. Since peptidergic knockdown of *itpr* phenocopies the *itpr*^*ku*^ mutant, transcript levels of the gastric lipase *magro* were measured from isolated guts of appropriate genotypes (Figure 5C). *magro* expression in guts from flies with knockdown of the IP_3_R in peptidergic neurons appears significantly elevated. Moreover, expression of *itpr*^*+*^ in peptidergic neurons of *itpr*^*ku*^ animals could rescue the elevated levels of *magro* transcripts (Figure 5C). Thus, IP_3_R mediated signals in peptidergic neurons appear to regulate *magro* transcription in the gut in a manner that is not yet understood.

## Discussion and conclusions

Insulin-like peptides (ILPs), which are secreted by a subset of the medial neurosecretory cells in the brain (Figure 1), regulate lipid homeostasis in the fat body cells of adult *Drosophila*[22,23]. The obese phenotype observed in adult *itpr* mutants suggested a role for IP_3_ mediated calcium signaling in modulating ILP release and secretion. However, significant differences were observed between the phenotypes of *itpr* mutant animals rescued by expression of an *itpr*^*+*^ cDNA as compared with rescue by over-expression of *Drosophila* ILP2, suggesting that IP_3_R mutants affect a broader axis of neurohormonal control than the one defined by insulin signaling [9]. IP_3_-mediated calcium signaling and its modulation of the neurohormonal axis, leading to obesity, have now been investigated in greater detail. The ILP secreting medial neurosecretory cells also secrete a number of other neuropeptides, which regulate stress and metabolism in *Drosophila*[24,25]. While it is possible that these neuropeptides, in addition to the ILPs, contribute to the *itpr* mutant phenotype of obesity this seems unlikely because knockdown of the IP_3_R in neurons which secrete ILPs and additional neuropeptides (the DILP neurons) had no effect on starvation resistance and obesity in *itpr* mutants. The peptidergic neurons, defined by *dimmGAL4*, include the DILP neurons plus other neurosecretory cells in the brain [18]. Because knockdown of the IP_3_R in peptidergic neurons phenocopied the *itpr* mutant, and restoring *itpr* function specifically in peptidergic neurons rescued mutant phenotypes, we conclude that IP_3_R function and Ca^2+^ release affects lipid metabolism primarily through regulation of neuropeptide secretion. The composite phenotypes of hyperphagia and obesity in IP_3_R mutants and peptidergic knockdown animals suggest a role for these neuropeptides in regulation of feeding and TAG storage and utilisation in the fatbody. Our data support the idea that feeding and lipid mobilisation are regulated by non-overlapping sets of neurosecretory cells, possibly comprising the mNSCs which regulate TAG utilisation in the fatbody, and other neuropeptide secreting cells such as the neurons of the stomatogastric system, which regulate satiety and feeding. Moreover, it is likely that feedback mechanisms exist between these two groups of neurons such that knockdown of the IP_3_R in one set can be compensated by the other, as in the case of knockdown in the DILP neurons. A recent study revealed the role of drosulfakinin (DSK), a neuropeptide expressed in the DILP neurons in feeding and satiety [25]. The target cells of DSK are not known and in the light of our observations, we hypothesize that these might be peptidergic neurons of the stomatogastric nervous system. Indeed, axonal projections from the mNSCs have been shown to target the proventriculus and midgut in both larval and adult animals [19]. Thus, when IP_3_R knockdown affects both sets of neurons, as in *dimmGAL4* knockdown and in *itpr* mutants, it is likely that such feedback mechanisms are abrogated. As a consequence despite the existence of sufficient TAG stores in the fatbody the animals continue to feed excessively, leading to a further increase in TAG deposits and obesity.

The altered neurohormonal axis created by knockdown of *itpr* in peptidergic neurons, and in *itpr*^*ku*^ mutants, leads to non-cell autonomous effects such as up-regulation of the gastric lipase *CG5932 (magro)* which has been previously reported to be expressed mainly in the proventriculus and then delivered to the intestinal lumen [11]. Our data support the idea that *magro* transcription is regulated by neuropeptides released from the stomatogastric nervous system (SNS). Stimuli received by the SNS are likely to derive from elevated feeding and body TAG levels. A similar neurohormonal gut brain axis is known to exist in mammals in which the vagus nerve which innervates the gastro-intestinal tract plays a role in regulating feeding, satiety and nutrient absorption [15,26] and responds to the orexin neuropeptides [27]. A better understanding of signaling mechanisms that regulate interactions between these axes is likely to help in devising new therapeutic measures for human obesity.

## Methods

### *Drosophila* strains

*itpr* RNAi experiments utilized the *UAS-dsitpr* strain (1063R-2) obtained from the National Institute of Genetics, Kyoto, Japan. The *UAS*-*itprGFP* strain used was from Srikanth et al., 2006 [28]. *itpr*^*ka1091/ug3*^ (*itpr*^*ku*^) is a heteroallelic combination of single point mutants in the *itpr* gene that were generated in an EMS (ethyl methane sulfonate) screen [8]. The embryonic wild-type *itpr* cDNA (*UASitpr*^*+*^) [5] was used for rescue experiments. *UASmCD8-GFP (II)* used was obtained from the Bloomington Stock Centre, Bloomington, IN. GAL4 strains used were a fat body GAL4,*c729GAL4*[29], a Dilp2 neuronal GAL4,*Dilp2GAL4*[30], a pan-neuronal GAL4, *elav*^*C155*^*GAL4*[31] and a peptidergic neuron GAL4, *dimmGAL4*[32].

### Starvation assays

Flies were grown on normal food (80 g of corn flour, 20 g of D-Glucose, 40 g of sucrose, 8 g of agar and 15 g of yeast extract in a total volume of 1 litre). They were aged on the same food for 3 days and the starvation assay was carried out as described in [9].

### Immunohistochemistry

Adult brains of the specified genotypes were dissected and fixed in 4% paraformaldehyde. A rabbit anti-GFP primary antibody (1:10,000; #A6455, Molecular Probes, Eugene, USA) was used with a rabbit Alexa Fluor 488 secondary (1:400; #A1108) to probe for levels of *UASitprGFP*^*+*^. The samples were mounted in 60% glycerol and confocal images were acquired using an Olympus FV1000 Confocal Microscope and viewed using FV10-ASW 3.0 viewer (Olympus Corporation, Japan).

### Oil red staining of neutral lipids

For Oil Red O staining, guts were fixed in PBS containing 4% paraformaldehyde. They were incubated for 20 to 30 min in 0.1% Oil Red O (Sigma, St. Louis, USA), washed in PBS and mounted in 60% glycerol. Photographs were obtained on an Olympus BX60 Microscope with an Evolution VF camera (Media Cybernetics, Bethesda, USA).

### TAG assay

TAGs were estimated with a Triglyceride reagent kit (GPO-ESPAS, Ranbaxy Diagnostic Limited, India) in homogenates from *Drosophila* abdomens and were normalized to protein levels as described in [9]. Total protein was estimated from the same homogenate using a BCA kit from Sigma-Aldrich.

### Quantification of feeding assay

Freshly eclosed flies were collected and aged for 3 days on normal food, starved for 24 hours and transferred into vials containing 1.2% red dye (Chromotrope FB, Sigma), 1% agar and 100 mM sucrose. They were allowed to feed in a dark chamber for 2 hours post which the intake of red dye was monitored from abdominal lysates of three independent batches, as described in [9].

### RNA isolation and qRT-PCR

Total RNA was isolated from ~5 guts of the indicated genotypes dissected in PBS for each sample. RNA isolation was performed using TRIzol reagent (Invitrogen, Life technologies, Carlsbad, CA, USA) following manufacturer’s instructions. RNA was dissolved in nuclease free water and quantified using a NanoDrop machine (Thermo scientific, Wilmington, DE, USA), and the integrity was checked on a 1.5% TAE gel. Approximately 500 ng of RNA was used for cDNA preparation by Reverse Transcription as described in [7]. qPCR was performed on an ABI 7500 fast machine operated with ABI 7500 software using MESA GREEN qPCR Master MIX Plus for SYBR Assay I dTTP (Eurogentec, Belgium). Each qPCR experiment was repeated three times with independently isolated RNA samples at 1:10 dilution. *rp49* was used as the internal control. A melt curve was performed after the assay to check for specificity of the reaction. The fold change of gene expression in the mutant relative to wild-type was determined by the comparative DDCt method [33]. In this method the fold change is 2^-DDCt^ where DDCt = (Ct (target gene) –Ct (rp49))_mutant_ - (Ct (target gene) – Ct (rp49))_Wild type._

Sequences of the primer used are as follows:

Rp49: F- 5′-CGGATCGATATGCTAAGCTGT-3′ R- 5′-GCGCTTGTTCGATCCGTA-3′

*CG5932*: *F-5′-GCAGCACGGATTGTTCAGTAA-3′ R-5′-CTGTTCAGCGAGATGATGATG-3′*

### Statistical analysis

Computations of means, standard error of the mean (SEM) and students t-tests were performed using Origin 7.5. All error bars in all figures represent SEM.

## Competing interests

The authors declare that they have no competing interests.

## Authors’ contributions

MS conducted the starvation and body weight experiments, TAG assay for the rescue, feeding assays and assembled the figures; SJ carried out the confocal imaging, feeding assays and helped in drafting of the manuscript; SR performed the transcript analysis, analyzed the data and drafted the manuscript; GH conceived the experiments, interpreted the results and critically revised the paper. All authors read and approved the final manuscript.
